# County‐level vulnerability is associated with mental health and substance use treatment among rural suicide decedents: A national multi‐year cross‐sectional study

**DOI:** 10.1111/jrh.70094

**Published:** 2025-11-06

**Authors:** J Priyanka Vakkalanka, Victor A Soupene, Jennifer Van Tiem, James M Blum, Barbara St. Marie

**Affiliations:** ^1^ Department of Emergency Medicine Carver College of Medicine, University of Iowa Iowa City Iowa USA; ^2^ Department of Epidemiology College of Public Health, University of Iowa Iowa City Iowa USA; ^3^ Department of Occupational and Environmental Health College of Public Health, University of Iowa Iowa City Iowa USA; ^4^ Office of Rural Health Veterans Rural Health Resource Center Iowa City VA Health Care System Iowa City Iowa USA; ^5^ Department of Family and Community Medicine Carver College of Medicine, University of Iowa Iowa City Iowa USA; ^6^ Health Care Information Systems University of Iowa Health Care Iowa City Iowa USA; ^7^ Department of Anesthesia University of Iowa Iowa City Iowa USA; ^8^ Institute for Clinical and Translational Science University of Iowa Iowa City Iowa USA; ^9^ College of Nursing University of Iowa Iowa City Iowa USA

**Keywords:** health, health care disparities rural, health services accessibility, social determinants of health, suicide

## Abstract

**Purpose:**

To examine the relationship between individual‐ and county‐level factors associated with mental health and substance use dependence (MHSUD) treatment among rural suicide decedents.

**Methods:**

Cross‐sectional study (2013–2022) study of the National Violent Death Reporting System and the County Health Rankings. Primary exposures included individual‐ (demographic, clinical conditions) and county‐level (average number of mentally unhealthy days, percentage of uninsured adults, rate of mental health providers/county, percentage of unemployed adults, rate of social associations, percentage of adults driving alone during long commutes, rate of primary care physicians/county, and income inequality ratios) factors of the decedent. The outcome was ever receipt of MHSUD treatment. We used multivariable logistic regression to measure the association between individual‐ and county‐level factors and MHSUD treatment receipt.

**Results:**

Of 42,021 rural suicides, 30% had MHSUD treatment receipt. Decedent‐level factors associated with lower MHSUD treatment included male, sex, older age, racial/ethnic minorities, and residence in the Midwest or Northeast. MHSUD treatment was lower in rural counties with greater vulnerability (e.g., higher average number of mentally unhealthy days [aOR = 0.75, 95% CI: 0.68, 0.81], lower rate of primary care physicians/county [aOR = 0.92, 95% CI: 0.85, 0.99], lower rate of mental health providers/county [aOR = 0.76, 95% CI: 0.70, 0.81]).

**Conclusions:**

By focusing within rural US counties, we found considerable variability in county‐level risk factors for MHSUD treatment among suicide decedents. Research and public health efforts may consider disaggregating county‐level factors when tailoring rural suicide prevention interventions in addition to improving MHSUD clinical infrastructure for both vulnerable individuals and counties.

## INTRODUCTION

Rural communities face heightened rates of untreated mental health and substance use disorder (MHSUD) compared to urban communities, driven by common structural, physical, and social barriers to availability and utilization.^1^, ^2^, ^3^, ^4^ Contributing to these disparities are the limited access to providers, especially MHSUD‐trained providers, and  transportation barriers.^1^ Stigma, isolation, and cultural beliefs create social barriers within rural settings and may further diminish MHSUD treatment engagement and utilization.^3^, ^4^, ^5^, ^6^, ^7^, ^8^, ^9^ While rural communities often share these systemic obstacles, broad definitions of *rural* can conceal important local variability in treatment utilization. This variability may then influence MHSUD outcomes in distinct ways, including the concerning uneven rise in rural suicide rates.^10^, ^11^

Prior research on the etiology and epidemiology of suicide has contributed to the development of a socioecological framework specifically for suicide risk factors and prevention.^12^ From this multi‐level perspective and framework, it is evident that individual,^13^, ^14^, ^15^, ^16^ interpersonal,^17^ community,^13^, ^18^, ^19^, ^20^ and societal levels contribute to suicide outcomes. Additionally, it has been established in the literature that treatment availability and utilization of services, which are linked to suicide outcomes as a potential contributing factor,^21^, ^22^ differ between urban and rural residents.^4^, ^14^, ^20^, ^23^, ^24^, ^25^, ^26^ Yet there remains a critical gap in isolating both individual‐ and county‐level factors specifically within rural contexts, as *rural* is broadly captured and does not account for variability. This gap constrains our understanding of (1) how differences in MHSUD treatment histories among rural residents and communities directly influence suicide risk, and (2) the diverse ways rural communities experience and address MHSUD‐related morbidity and mortality.

We aimed to examine the associations between individual and county health rankings, and the history of MHSUD treatment among rural suicide decedents, using the Social Ecological Model for Suicide Risk and Prevention. This model helped us consider how factors at multiple levels contribute to suicide risk and treatment engagement, even when isolating suicide deaths within the rural setting. We also explore variability in county‐level health rankings across urban and rural settings, with particular attention to rural‐specific differences in MHSUD treatment history. By grounding our analysis in this multi‐level framework, we seek to inform targeted and contextually relevant prevention strategies, guide future research priorities, and support a more equitable allocation of MHSUD resources in rural communities.

## METHODS

### Study design, setting, sample

We conducted a multi‐year (2013–2022) cross‐sectional study of rural suicide deaths reported to the National Violent Death Reporting System (NVDRS). The NVDRS is a case‐only, population‐based surveillance system of violence‐related mortality (e.g., suicides).^27^ Data from the NVDRS comes from death certificates, law enforcement reports, and coroner/medical examiner reports. The NVDRS was tracking violent deaths for all 50 US states, the District of Columbia, and Puerto Rico by 2020. Because we are using data during 2013–2022, we included data when made available (see Appendix A for states contributing to the NVDRS by year).^27^, ^28^ The second data source we used included the University of Wisconsin's County Health Rankings^29^ classification of county‐level characteristics, linked to resident codes from NVDRS to determine rurality from the 2023 rural‐urban continuum codes (RUCC).^30^, ^31^ County health rankings were designed to provide measures related to health like clinical care, social and economic factors, and physical environment for nearly all US counties.^32^ We included both urban and rural suicide decedents for the descriptive assessment of county health rankings. However, only suicides that occurred in rural counties (i.e., decedents from counties with RUCCs of 4 to 9) were included for primary analysis assessing rural‐specific differences related to MHSUD treatment history. As per NVDRS guidance,^27^, ^33^ we excluded decedents with unknown circumstances and those with missing county‐level information from the University of Wisconsin's County Health Rankings, for the RUCC data. This study was deemed non‐human subjects research by the local Institutional Review Board, and is reported in accordance with the Strengthening the Reporting of Observational Studies in Epidemiology (STROBE) guidelines.^34^

### Study measures

Data in NVDRS includes information such as demographics, mental health conditions and history, and treatment. The outcome washaving a known history of MHSUD treatment reported from next‐of‐kin, law enforcement, or medical examiner reports dichotomized as yes or no.^27^ At the decedent‐level, we included sex, age group, race/ethnicity, education level, marital status, military affiliation, and region of residence. Age was categorized as 18–24, 25–44, 45–64, and 65+ years. Region was categorized as Midwest, west, northeast and southeast from the US Census Bureau.^35^ From NVDRS, we captured use of lethal means, categorized as firearm, poisoning, asphyxiation, and other.

To operationalize community‐level factors in suicide risk and prevention, we included several county health rankings determined from previous literature to be associated with suicide and access to mental health treatment.^36^, ^37^, ^38^ These county‐level characteristics included average number of mentally unhealthy days, percentage of uninsured adults, rate of mental health providers per county, percentage of unemployed adults, rate of social associations, percentage of adults driving alone during a long commute, rate of primary care physicians per county, and income inequality ratios were considered. We used the County Health Rankings’ classification of these characteristics in quartiles with the lowest number (smallest ratio) corresponding to the first quartile and the highest number (largest ratio) corresponding to the fourth quartile.

### Statistical analysis

We descriptively (medians, interquartile ranges [IQR]) reported on county health rankings separately for urban and rural suicide decedents (Appendix B). Then among rural suicide decedents only, we compared all individual‐ and county‐level characteristics between those with and without a history of MHSUD treatment using logistic regression models. We developed a multivariable logistic regression model to examine how county‐level factors contributed to a reported history of receiving MHSUD treatment compared to those who do not, adjusted for age group, sex, race/ethnicity, and education level. Though we acknowledge that there may be shared attributes within counties, we did not cluster this analysis on county as there were over 1,000 counties represented in the dataset. We built models using forward selection with Akaike Information Criterion (AIC) and report unadjusted (uOR) and adjusted (aOR) odds ratios with 95% confidence intervals (CI) for each variable. We conducted all analyses using SAS (SAS Institute Inc, Cary, NC).

## RESULTS

Descriptive aspects of county health rankings among rural residents are presented in Table 1. While there were narrower distributions across several measures among rural suicide decedents, the median rate of primary care providers per county among rural decedents was 51 per 100,000 residents and ranged from 37 per 100,000 (25th percentile) to 70 per 100,000 (75th percentile). Similarly, the median rate of mental health providers per county was 164 per 100,000 residents across rural communities but ranged from 90 per 100,000 (25th percentile) and 294 per 100,000 (75th percentile).

**TABLE 1:** County‐level characteristics among rural US suicide decedents.

	Rural US decedents (*n* = 42,021)
County‐level characteristics	Median (IQR)
Average number of mentally unhealthy days	4.87 (4.42–5.22)
Percentage of uninsured adults	10.03 (7.57–13.81)
Rate of primary care physicians	50.74 (37.46–70.45)
Rate of population to mental health providers	163.66 (90.15–293.75)
Percentage of unemployed adults	3.99 (3.28–4.90)
Income inequality ratio^1^	4.39 (4.06–4.79)
Rate of social associations	11.81 (9.28–14.35)
Percentage of adults driving alone during a long commute	29.00 (20.80–35.40)

^1^ Income inequality ratio was calculated as the 80th income percentile/the 20th income percentile.

Of the 42,021 suicide decedents who died in the rural United States during 2013–2022 in the analytical sample (Appendix C), 30.3% (*n* = 12,729) had a history of MHSUD treatment (Table 2). From the unadjusted model results, nearly all individual‐level demographic characteristics were associated with a history of MHSUD treatment among rural suicide decedents. Compared to suicide deaths by asphyxiation, use of a firearms as a lethal means was associated with lower MHSUD treatment history (uOR = 0.71, 95% CI: 0.68–0.75), while MHSUD treatment history was greater among those with poisoning identified as the lethal means (uOR: 1.89; 95% CI: 1.76–2.03). At the county‐level, nearly all factors were associated with a history of MHSUD treatment.

**TABLE 2:** Characteristics by history of mental health or substance use treatment use among rural US suicide decedents.

	All (*n* = 42,021)		History of MHSUD treatment (*n* = 12,729)		No history of MHSUD treatment (*n* = 29,292)		Unadjusted OR (95% CI)
Characteristics	*N*	%	*N*	%	*N*	%	
***Individual‐level characteristics***							
*Sex*							
Male	33,829	80.5	9077	71.3	24,752	84.5	**0.46 (0.43–0.48)**
Female	8192	19.5	3652	28.7	4540	15.5	Ref
*Age group*							
18–24	4110	9.8	1169	9.2	2941	10.0	**0.77 (0.70–0.84)**
45–64	14,137	33.6	4811	37.8	9326	31.8	**1.24 (1.17–1.31)**
65+	10,165	24.2	2429	19.1	7736	26.4	**0.83 (0.78–0.89)**
25–44	13,609	32.4	4320	33.9	9289	31.7	**Ref**
*Race/ethnicity*							
Black, NH	1043	2.5	261	2.1	782	2.7	**0.74 (0.65–0.86)**
Asian/Pacific Islander	254	0.6	58	0.5	196	0.7	**0.66 (0.49–0.89)**
Hispanic	1698	4.0	448	3.5	1250	4.3	**0.80 (0.72–0.89)**
Other, NH	1910	4.6	467	3.7	1443	4.9	**0.72 (0.65–0.80)**
White, NH	37,116	88.3	11,495	90.3	25,621	87.5	Ref
*Education level*							
High school graduate or less	26,495	66.9	7245	61.2	19,250	69.3	**0.67 (0.60–0.75)**
Some college or associate's degree	8650	21.8	2934	24.8	5716	20.6	0.92 (0.82–1.03)
Bachelor's degree	3059	7.7	1159	9.8	1900	6.8	1.09 (0.96–1.25)
Master's, doctorate, or professional degree	1401	3.5	502	4.2	899	3.2	Ref
*Marital status*							
Never married	13,292	31.9	4060	32.1	9232	31.8	0.98 (0.93–1.03)
Single, not otherwise specified	855	2.1	234	1.9	621	2.1	**0.84 (0.72–0.98)**
Divorced	9565	23.0	2914	23.1	6651	22.9	0.98 (0.92–1.03)
Widowed	2817	6.8	648	5.1	2169	7.5	**0.67 (0.61–0.73)**
Married/civil union/domestic partnership, but separated	1360	3.3	514	4.1	846	2.9	**1.35 (1.21–1.52)**
Married/civil union/domestic partnership	13,779	33.1	4272	33.8	9507	32.8	Ref
*Region*							
Midwest	13,514	32.3	4130	32.5	9384	32.1	1.01 (0.96–1.06)
West	9020	21.5	2608	20.5	6412	21.9	0.93 (0.88–1.15)
Northeast	3636	8.7	1159	9.1	2477	8.5	1.07 (0.99–1.15)
Southeast	15,757	37.6	4800	37.8	10,957	37.5	Ref
*Lethal mean used*							
Firearm	25,095	59.7	6399	50.3	18,696	63.8	**0.71 (0.68–0.75)**
Poisoning	4644	11.1	2214	17.4	2430	8.3	**1.89 (1.76–2.03)**
Other	1918	4.6	747	5.9	1171	4.0	**1.33 (1.20–1.47)**
Asphyxiation	10,364	24.7	3369	26.5	6995	23.9	Ref
*Veteran status*							
Yes	7289	17.36	1879	14.77	5410	18.49	**0.76 (0.72–0.80)**
Unknown	774	1.84	197	1.55	577	1.97	**0.75 (0.63–0.88)**
No	33,922	80.80	10,643	83.68	23,279	79.54	Ref
***County‐level characteristics***							
*Average number of mentally unhealthy days* ^a^							
1st	10,517	25.03	3567	28.02	6950	23.73	Ref
2nd	10,599	25.22	3330	26.16	7269	24.82	**0.89 (0.84–0.95)**
3rd	10,394	24.74	3036	23.85	7358	25.12	**0.80 (0.76–0.85)**
4th	10,511	25.01	2796	21.97	7715	26.34	**0.71 (0.67–0.75)**
*Percentage of uninsured adults* ^a^							
1st	10,562	25.14	3401	26.72	7161	24.45	Ref
2nd	10,507	25.00	2946	23.14	7561	25.81	**0.82 (0.77–0.87)**
3rd	10,460	24.89	3474	27.29	6986	23.85	1.05 (0.99–1.11)
4th	10,492	24.97	2908	22.85	7584	25.89	**0.81 (0.76–0.86)**
*Rate of primary care physicians* ^a^							
1st	10,932	26.02	3067	24.09	7865	26.85	**0.83 (0.78–0.88)**
2nd	10,356	24.64	3181	24.99	7175	24.49	**0.94 (0.88–0.99)**
3rd	10,390	24.73	3162	24.84	7228	24.68	**0.93 (0.87–0.98)**
4th	10,343	24.61	3319	26.07	7024	23.98	Ref
*Rate of mental health providers* ^a^							
1st	11,032	26.25	2936	23.07	8096	27.64	**0.81 (0.77–0.86)**
2nd	10,327	24.58	3160	24.83	7167	24.47	0.99 (0.93–1.05)
3rd	10,632	25.30	3537	27.79	7095	24.22	1.12 (1.05–1.18)
4th	10,030	23.87	3096	24.32	6934	23.67	Ref
*Percentage of unemployed adults* ^a^							
1st	10,574	25.16	3247	25.51	7327	25.01	–
2nd	10,475	24.93	3372	26.49	7103	24.25	**1.07 (1.01–1.14)**
3rd	10,478	24.94	3270	25.69	7208	24.61	1.02 (0.97–1.09)
4th	10,494	24.97	2840	22.31	7654	26.13	**0.84 (0.79–0.89)**
*Income inequality ratio* ^a^, ^b^							
1st	10,576	25.17	3472	27.28	7104	24.25	Ref
2nd	10,478	24.94	3246	25.50	7232	24.69	**0.92 (0.87–0.97)**
3rd	10,518	25.03	3259	25.60	7259	24.78	**0.92 (0.87–0.97)**
4th	10,449	24.87	2752	21.62	7697	26.28	**0.73 (0.69–0.78**)
*Rate of social associations* ^a^							
1st	10,498	24.98	2898	22.77	7600	25.95	**Ref**
2nd	10,618	25.27	3165	24.86	7453	25.44	**1.11 (1.05–1.18)**
3rd	10,434	24.83	3229	25.37	7205	24.60	**1.18 (1.11–1.25)**
4th	10,471	24.92	3437	27.00	7034	24.01	**1.28 (1.21–1.36)**
*Percentage of adults driving alone during a long commute* ^a^							
1st	10,787	25.67	3185	25.02	7602	25.95	Ref
2nd	10,359	24.65	3077	24.17	7282	24.86	1.01 (0.95–1.07)
3rd	10,457	24.89	3389	26.62	7068	24.13	**1.14 (1.08–1.21)**
4th	10,418	24.79	3078	24.18	7340	25.06	1.00 (0.94–1.06)

*Note*: Bold value indicates *p* < 0.05.

Abbreviations: MH = mental health; NH = non‐Hispanic; SU = substance use.

^a^ For county‐level characteristics: 1st = lowest number, 4th = highest number.

^b^ Income Inequality ratio was calculated as the 80th income percentile/the 20th income percentile.

In adjusted models, several individual‐level factors were associated with a history of MHSUD treatment receipt. Rural suicide decedents who were less likely to have a history of MHSUD treatment included males (aOR = 0.54, 95% CI: 0.51–0.57) compared to females; 65+ years (aOR = 0.80, 95% CI: 0.74–0.85) compared to 25–44 years; all races/ethnicities compared to non‐Hispanic Whites; high school graduates or less (aOR = 0.74, 95% CI: 0.66–0.83) compared to those with a bachelor's degree; those living in the Midwest (aOR = 0.85, 95% CI: 0.79–0.91), West (aOR = 0.80, 95% CI: 0.73–0.87), or Northeast (aOR = 0.84, 95% CI: 0.76–0.92) compared to the Southeast; and those who used a firearm in their death (aOR = 0.74, 95% CI: 0.70–0.78) compared to those who died from asphyxiation (Table 3).

**TABLE 3:** The association between individual‐ and county‐level characteristics and having a history of MHSUD treatment use among rural US suicide decedents.

Characteristics	*N*	Adjusted OR (95% CI)^a^
**Individual‐level characteristics**		
*Sex*		
Male	33,829	**0.54 (0.51–0.57)**
Female	8192	Ref
*Age group*		
18–24	4110	0.95 (0.87–1.03)
45–64	14,137	**1.08 (1.02–1.14)**
65+	10,165	**0.80 (0.74–0.85)**
25–44	13,609	Ref
*Race/ethnicity*		
Black, NH	1043	**0.73 (0.62–0.85)**
Asian/Pacific Islander	254	**0.54 (0.40–0.74)**
Hispanic	1698	**0.82 (0.72–0.93)**
Other, NH	1910	**0.78 (0.69–0.88)**
White, NH	37,116	Ref
*Education level*		
High school graduate or less	26,495	**0.74 (0.66–0.83)**
Some college or associate's degree	8650	0.91 (0.81–1.03)
Bachelor's degree	3059	1.08 (0.94–1.24)
Master's, doctorate, or professional degree	1401	Ref
*Marital status*		
Never married	13,292	1.02 (0.96–1.09)
Single, not otherwise specified	855	0.91 (0.77–1.08)
Divorced	9565	**0.87 (0.82–0.93)**
Widowed	2817	**0.69 (0.62–0.77)**
Married/civil union/domestic partnership, but separated	1360	**1.19 (1.05–1.35)**
Married/civil union/domestic partnership	13,779	Ref
*Region*		
Midwest	13,514	**0.85 (0.79–0.91)**
West	9020	**0.80 (0.73–0.87)**
Northeast	3636	**0.84 (0.76–0.92)**
Southeast	15,757	Ref
*Lethal mean used*		
Firearm	25,095	**0.74 (0.70–0.78)**
Poisoning	4644	**1.46 (1.35–1.58)**
Other	1918	**1.24 (1.11–1.38)**
Asphyxiation	10,364	Ref
*Veteran status*		
Yes	7289	0.98 (0.92–1.05)
Unknown	774	**0.74 (0.60–0.92)**
No	33,922	Ref
**County‐level characteristics**		
*Average number of mentally unhealthy days* ^b^		
1st	10,517	Ref
2nd	10,599	**0.83 (0.77–0.89)**
3rd	10,394	**0.78 (0.72–0.84)**
4th	10,511	**0.75 (0.69–0.82)**
*Percentage of uninsured adults* ^b^		
1st	10,562	Ref
2nd	10,507	**0.90 (0.84–0.96)**
3rd	10,460	**1.15 (1.07–1.23)**
4th	10,492	0.92 (0.85–1.00)
*Rate of primary care physicians* ^b^		
1st	10,932	**0.92 (0.85–0.99)**
2nd	10,356	1.02 (0.96–1.10)
3rd	10,390	0.98 (0.92–1.05)
4th	10,343	Ref
*Rate of mental health providers* ^b^		
1st	11,032	**0.75 (0.69–0.81)**
2nd	10,327	**0.91 (0.84–0.97)**
3rd	10,632	1.01 (0.95–1.08)
4th	10,030	Ref
*Percentage of unemployed adults* ^b^		
1st	10,574	Ref
2nd	10,475	**1.12 (1.05–1.20)**
3rd	10,478	**1.15 (1.08–1.24)**
4th	10,494	**1.10 (1.02–1.19)**
*Income inequality ratio* ^a^, ^c^		
1st	10,576	Ref
2nd	10,478	**0.87 (0.81–0.93)**
3rd	10,518	**0.89 (0.83–0.95)**
4th	10,449	**0.74 (0.68–0.80)**
*Rate of social associations* ^b^		
1st	10,498	Ref
2nd	10,618	1.04 (0.97–1.11)
3rd	10,434	1.06 (0.98–1.14)
4th	10,471	**1.14 (1.06–1.23)**
*Percentage of adults driving alone during a long commute* ^b^		
1st	10,787	Ref
2nd	10,359	1.03 (0.96–1.10)
3rd	10,457	**1.18 (1.09–1.26)**
4th	10,418	**1.11 (1.03–1.20)**

^a^ Adjusted for age group, sex, race/ethnicity, and education level. Bolded *p*‐values < 0.05.

^b^ For county‐level characteristics: 1st = lowest number, 4th = highest number.

^c^ Income Inequality ratio was calculated as the 80th income percentile/the 20th income percentile.

We observed community‐level factors associated a history of treatment receipt, and we briefly describe the comparison between the 1st and 4th quartiles here. History of MHSUD treatment was lower in counties with a higher average number of mentally unhealthy days (aOR = 0.75, 95% CI: 0.69–0.82), a higher percentage of uninsured adults (aOR = 0.92, 95% CI: 0.85–1.00), a lower rate of primary care physicians per county (aOR = 0.92, 95% CI: 0.85–1.00), a lower rate of mental health providers per county (aOR = 0.75, 95% CI: 0.69–0.81), and a higher income inequality ratio (aOR = 0.74, 95% CI: 0.68–0.80) (Table 3). A history of MHSUD treatment was greater among those with higher rates of social associations (aOR = 1.14, 95% CI: 1.06–1.23) and higher percentage of adults driving alone during a long commute (aOR = 1.11, 95% CI: 1.03–1.20).

## DISCUSSION

In this multiyear, cross‐sectional study of rural suicides, we observed both individual‐level and community‐level factors are associated with documented MHSUD treatment. These findings suggest that (1) despite shared characteristics, variability within rural settings may contribute to MHSUD treatment and suicide; (2) community‐level structural barriers such as policies and practices related to rural populations may prevent or reduce access to health care; and (3) individual‐level demographic factors nested within these community‐level factors including age, sex, race/ethnicity, and education level still contribute to underutilization of MHSUD treatment independently. Together, these findings underscore the importance of addressing suicide prevention through a, recognizing that both individual and structural determinants must be considered to effectively improve MHSUD treatment access and reduce suicide risk in rural communities.

While we observed that only 30% of rural suicide decedents sought prior MHSUD treatment, there was still variability across community‐level factors including social and economic‐related indicators within rural areas. For example, suicide decedents in rural areas with higher rates of mentally unhealthy days, uninsured residents and unemployment, and lower availability of mental health providers experienced lower likelihood of documented MHSUD treatment. In contrast, decedents residing in rural counties with higher rates of social associations were associated with an increased likelihood of having a history of MHSUD treatment. These findings, in aggregate, are consistent with previous clinical and policy research that suggests geographical, community, and societal differences in MHSUD risk and treatment across the rural spectrum.^5^, ^39^, ^40^, ^41^, ^42^ There may also be a need for policy‐related interventions or programs that address broader problems such as limited health literacy, lower coverage through insurance, stigma, and recognition of the need for preventative and screening services. For example, lower health literacy may reduce awareness of treatment options, hinder navigation of the health care system, and lead to decreased adherence with medications or treatment plans.^43^, ^44^ By improving health literacy, individuals in rural communities may be better equipped to increase engagement with health care providers and services. In parallel, addressing stigma surrounding mental health and substance use in rural areas remains critical overall, and especially among rural males.^45^, ^46^ Stigma can discourage individuals from seeking help, reduce community support for mental health initiatives, and perpetuate silence around suicide risk.^47^ Culturally responsive, community‐based efforts that normalize help‐seeking as a shared responsibility may be essential for reducing stigma and fostering environments where individuals feel comfortable accessing MHSUD care.

Our findings may also suggest that variability in structural barriers can prevent access to care even where services exist in rural areas. For example, we observed individual‐level demographic risk factors (i.e., male sex, older age, non‐white, and less educated decedents) were associated with decreased history of MHSUD treatment, even when nested in communities with better availability of services. Though we did not examine this, findings from previous work suggest that there is fear of being recognized at mental health clinics,^41^ perception in rural communities of the importance of self‐reliance, and a mistrust of systems,^48^ leading rural residents to avoid seeking services. While these findings have been previously established in the literature,^49^, ^50^ it is also possible that the combination of these demographic factors within communities of greater socioeconomic vulnerability (i.e., individual risk factors nested within community‐level risk factors of the Social Ecological Model for Suicide) can synergistically lead to worse outcomes. Independent individual risk factors that are also adjusted for community‐level factors may also suggest a potential mismatch in availability versus utilization. This aspect was particularly highlighted after the onset of the COVID‐19 public health emergency when care for MHSUD via telehealth increased widely in availability, yet availability did not translate to access and utilization of services across key groups, including rural and older residents.^51^

There were two key limitations to this study. First, the NVDRS is a public surveillance tool that may be subject to under‐reporting or availability of documentation of circumstances or clinical risk factors, particularly in rural areas.^52^ This may have led to some attenuation of observed findings. Second, since NVDRS is a case‐based surveillance system that allows for cross‐sectional analysis, we cannot establish any causal relationships between the observed risk factors and MHSUD treatment history among suicide decedents. Third, we did not cluster on counties in our models due to large number counties and the sparseness of data within them. While we directly included the county‐level characteristics into the models, this approach would not fully account for potential clustering effects.

In conclusion, rural communities are not homogenous, as the evidence suggests that MHSUD risk factors, treatment access, and county‐level factors differ widely across the rural spectrum, potentially due to geographic, demographic, policy, and infrastructure variability. Clinical research efforts for rural suicide prevention should consider both individual and county‐level disparities across the rural United States. Future research should explore causal mechanics and longitudinal trends and consider individual and county‐level factors when developing interventions for rural populations.

## CONFLICT OF INTEREST STATEMENT

All authors declare no conflicts of interest.

## DISCLAIMER

The findings and conclusions of this study are those of the authors alone and do not necessarily represent the official position of the CDC or of participating NVDRS states. During the preparation of this work, the authors used ChatGPT for portions of the text in order to review grammar and improve the clarity. After using this tool/service, the authors reviewed and edited the content as needed and take full responsibility for the content of the published article.

## Supporting information



Appendix A. States contributing to the NVDRS by year, 2013–2022

Appendix B. County‐level Characteristics by rurality of residence among US suicide decedents

Appendix C. Flow Chart OF Analytical Sample, NVDRS 2013–2022

## Data Availability

Data from the National Violent Death Reporting System Restricted Access Database are available upon request from the Centers for Disease Control and Prevention.

## References

[1] Browne T , Priester MA , Clone S , Iachini A , DeHart D , Hock R . Barriers and facilitators to substance use treatment in the rural south: a qualitative study. J Rural Health. 2016;32(1):92‐101. 26184098 10.1111/jrh.12129

[2] Heitkamp TL , Fox LF . Addressing disparities for persons with substance use disorders in rural communities. J Addict Nurs. 2022;33(3):191‐197. 36041162 10.1097/JAN.0000000000000483

[3] Morales DA , Barksdale CL , Beckel‐Mitchener AC . A call to action to address rural mental health disparities. J Clin Transl Sci. 2020;4(5):463‐467. 33244437 10.1017/cts.2020.42 PMC7681156

[4] Afifi RA , Parker EA , Dino G , Hall DM , Ulin B . Reimagining rural: shifting paradigms about health and well‐being in the Rural United States. Annu Rev Public Health. 2022;43:135‐154. 34910581 10.1146/annurev-publhealth-052020-123413 PMC11295601

[5] Andrilla CHA , Patterson DG , Garberson LA , Coulthard C , Larson EH . Geographic variation in the supply of selected behavioral health providers. Am J Prev Med. 2018;54(6):S199‐S207. Supplement 3. 29779543 10.1016/j.amepre.2018.01.004

[6] Borders TF , Booth BM . Research on rural residence and access to drug abuse services: where are we and where do we go? J Rural Health. 2007;23:79‐83. 18237329 10.1111/j.1748-0361.2007.00128.x

[7] RHIhub.Rural Mental Health. Available at: Accessed October 28, 2025. https://www.ruralhealthinfo.org/topics/mental‐health

[8] Rossen LM , Khan D , Warner M . Hot spots in mortality from drug poisoning in the United States. Health Place. 2014;26:14‐20. 2007‐2009. 24333939 10.1016/j.healthplace.2013.11.005 PMC4659494

[9] Weeks WB , Chang JE , Pagán JA , et al. Rural‐urban disparities in health outcomes, clinical care, health behaviors, and social determinants of health and an action‐oriented, dynamic tool for visualizing them. PLOS Glob Public Health. 2023;3(10):e0002420. 37788228 10.1371/journal.pgph.0002420 PMC10547156

[10] Mohatt NV , Kreisel CJ , Hoffberg AS , Mph LW , Beehler SJ . A systematic review of factors impacting suicide risk among rural adults in the United States. J Rural Health. 2021;37(3):565‐575. 33210399 10.1111/jrh.12532

[11] Casant J , Helbich M . Inequalities of suicide mortality across urban and rural areas: a literature review. Int J Environ Res Public Health. 2022;19(5):2669. 35270369 10.3390/ijerph19052669 PMC8909802

[12] Ullman K , Landsteiner A , Linskens E , et al. Risk and protective factors across socioecological levels of risk for suicide: an evidence map. Washington (DC): Department of Veterans Affairs (US); August 2021. https://www.ncbi.nlm.nih.gov/books/NBK575587/ 34846828

[13] Acevedo A , Panas L , Garnick D , et al. Disparities in the treatment of substance use disorders: does where you live matter? J Behav Health Serv Res. 2018;45(4):533‐549. 29435862 10.1007/s11414-018-9586-y PMC6087681

[14] Fontanella CA , Hiance‐Steelesmith DL , Phillips GS , et al. Widening rural‐urban disparities in youth suicides, United States, 1996‐2010. JAMA Pediatrics. 2015;169(5):466. 25751611 10.1001/jamapediatrics.2014.3561 PMC4551430

[15] National Institute of Mental Health. Suicide. Accessed October 28, 2025. https://www.nimh.nih.gov/health/statistics/suicide

[16] Centers for Disease Control and Prevention. Suicide Data and Statistics. Accessed October 28, 2025. https://www.cdc.gov/suicide/facts/data.html

[17] Cramer RJ , Kapusta ND . A social‐ecological framework of theory, assessment, and prevention of suicide. Front Psychol. 2017;8:1756. 29062296 10.3389/fpsyg.2017.01756 PMC5640776

[18] Mengell KA , Chikawa MMN , Weinstein JN , Welch R , Smallwood SW , Hansen AR . A systematic review of rural community‐based mental health interventions in the United States. J Ment Health. 2024:1‐10. 10.1080/09638237.2024.2361229 38835202

[19] Cummings JR , Allen L , Clennon J , Ji X , Druss BG . Geographic access to specialty mental health care across High‐ and Low‐Income US communities. JAMA Psychiatry. 2017;74(5):476. 28384733 10.1001/jamapsychiatry.2017.0303 PMC5693377

[20] Steelesmith DL , Fontanella CA , Campo JV , Bridge JA , Warren KL , Root ED . Contextual factors associated with county‐level suicide rates in the United States. JAMA Netw Open. 2019;2(9):e1910936. 1999 to 2016. 31490540 10.1001/jamanetworkopen.2019.10936 PMC6735416

[21] Hirsch JK , Cukrowicz KC . Suicide in rural areas: an updated review of the literature. J Rural Mental Health. 2014;38(2):65‐78.

[22] Luoma JB , Martin CE , Pearson JL . Contact with mental health and primary care providers before suicide: a review of the evidence. Am J Psychiatry. 2002;159(6):909‐916. 12042175 10.1176/appi.ajp.159.6.909 PMC5072576

[23] Raver E , Retchin SM , Li Y , Carlo AD , Xu WY . Rural‐urban differences in out‐of‐network treatment initiation and engagement rates for substance use disorders. Health Serv Res. 2024;59(5):e14299. 38456488 10.1111/1475-6773.14299 PMC11366955

[24] Franz B , Cronin CE , Lindenfeld Z , et al. Rural‐urban disparities in the availability of hospital‐based screening, medications for opioid use disorder, and addiction consult services. J Subs Use Addict Treat. 2024;160:209280. 10.1016/j.josat.2023.209280 PMC11060933 38142042

[25] RHIhub . Substance Use and Misuse in Rural Areas. Accessed October 28, 2025. https://www.ruralhealthinfo.org/topics/substance‐use#:~:text=Behavioral%20health%20and%20detoxification%20(detox,services%20for%20long%2Dterm%20recovery

[26] Soupene V , Hays T , Davis J , Vakkalanka JP . Factors associated with multiple death suicides among rural US decedents. J Rural Health. 2025;41(1):e70003. 39962337 10.1111/jrh.70003 PMC11832807

[27] National Violent Death Reporting System Web Coding Manual Version 6. Centers for Disease Control and Prevention, National Center for Injury Prevention and Control; 2022.

[28] National Violent Death Reporting System (NVDRS) . Centers for Disease Control and Prevention, National Centers for Injury Prevention and Control; 2022.

[29] University of Wisconsin Population Health Institute. County Health Rankings & Roadmaps. Accessed October 28, 2025. https://www.countyhealthrankings.org/health‐data/methodology‐and‐sources/methods#:~:text=Why%20are%20some%20counties%20not,is%20suppressed%20for%20privacy%20reasons

[30] Rural‐Urban Continuum Codes. Accessed October 28, 2025. https://www.ers.usda.gov/data‐products/rural‐urban‐continuum‐codes

[31] Rural‐Urban Continuum Codes . 2025. United States Department of Agriculture. Accessed October 28, 2025. https://www.ers.usda.gov/data‐products/rural‐urban‐continuum‐codes

[32] Hood CM , Gennuso KP , Swain GR , BB Catlin . County health rankings: relationships between determinant factors and health outcomes. Am J Prev Med. 2016;50(2):129‐135. 26526164 10.1016/j.amepre.2015.08.024

[33] Centers for Disease Control and Prevention. National Violent Death Reporting System: Web Coding Manual: Version 6.0 [2022‐01‐18]. Accessed October 28, 2025. https://stacks.cdc.gov/view/cdc/44789

[34] von Elm E , Altman DG , Egger M , Pocock SJ , Gøtzsche PC , Vandenbroucke JP . The Strengthening the Reporting of Observational Studies in Epidemiology (STROBE) statement: guidelines for reporting observational studies. J Clin Epidemiol. 2008;61(4):344‐349. 18313558 10.1016/j.jclinepi.2007.11.008

[35] National Center for Health Statistics, Centers for Disease Control and Prevention. Geographic Division or Region. Accessed October 28, 2025. https://www.cdc.gov/nchs/hus/sources‐definitions/geographic‐region.htm

[36] Tondo L , Albert MJ , Baldessarini RJ . Suicide rates in relation to health care access in the United States: an ecological study. Journal of Clinical Psychiatry. 2006;67(4):517‐523. 16669716 10.4088/jcp.v67n0402

[37] Hung P , Busch SH , Shih Y‐W , McGregor AJ , Wang S . Changes in community mental health services availability and suicide mortality in the US: a retrospective study. BMC Psychiatry. 2020;20(1):188. 32334552 10.1186/s12888-020-02607-y PMC7183673

[38] Ku BS , Li J , Lally C , Compton MT , Druss BG . Associations between mental health shortage areas and county‐level suicide rates among adults aged 25 and older in the USA. Gen Hosp Psychiatr. 2021;70:44‐50. 10.1016/j.genhosppsych.2021.02.001 PMC8127358 33714795

[39] Ziller EC , Anderson NJ , Coburn AF . Access to rural mental health services: service use and out‐of‐pocket costs. J Rural Health. 2010;26(3):214‐224. 20633089 10.1111/j.1748-0361.2010.00291.x

[40] Hirchak KA , Murphy SM . Assessing differences in the availability of opioid addiction therapy options: rural versus urban and American Indian reservation versus nonreservation. J Rural Health. 2017;33(1):102‐109. 26987797 10.1111/jrh.12178 PMC5568536

[41] Smalley KB , Yancey CT , Warren JC , Naufel K , Ryan R , Pugh JL . Rural mental health and psychological treatment: a review for practitioners. J Clin Psychol. 2010;66(5):479‐489. 20222125 10.1002/jclp.20688

[42] John G , Janis J , Coburn A , et al. Behavioral health in rural America: challenges and opportunities. Accesssed October 28, 2025. https://rupri.org/wp-content/uploads/Behavioral-Health-in-Rural-America-Challenges-and-Opportunities.pdf

[43] Miller TA . Health literacy and adherence to medical treatment in chronic and acute illness: a meta‐analysis. Patient Educ Couns. 2016;99(7):1079‐1086. 26899632 10.1016/j.pec.2016.01.020 PMC4912447

[44] Paasche‐Orlow MK , Wolf MS . addressing health literacy. Jama. 2025;333(20):1826. 40202765 10.1001/jama.2025.2198 PMC12435932

[45] Crumb L , Mingo TM , Crowe A . Get over it and move on″: the impact of mental illness stigma in rural, low‐income United States populations. Mental Health Prev. 2019;13:143‐148.

[46] Alang SM . Sociodemographic disparities associated with perceived causes of unmet need for mental health care. Psychiatr Rehab J. 2015;38(4):293‐299. 10.1037/prj0000113 25664758

[47] Corrigan PW , Druss BG , Perlick DA . The impact of mental illness stigma on seeking and participating in mental health care. Psychol Sci Public Inter. 2014;15(2):37‐70. 10.1177/1529100614531398 26171956

[48] Hoyt DR , Conger RD , Valde JG , Weihs K . Psychological distress and help seeking in rural America. Am J Community Psychol. 1997;25(4):449‐470. 9338954 10.1023/a:1024655521619

[49] Martikainen P , Mäki N , Blomgren J . The effects of area and individual social characteristics on suicide risk: a multilevel study of relative contribution and effect modification. Eur J Popul. 2004;20(4):323‐350.

[50] Jakobsen AL , Lund RL . Neighborhood social context and suicide mortality: a multilevel register‐based 5‐year follow‐up study of 2.7 million individuals. Soc Sci Med. 2022;311:115320. 36081301 10.1016/j.socscimed.2022.115320

[51] Vakkalanka JP , Gadag K , Lavin L , et al. Telehealth use and health equity for mental health and substance use disorder during the COVID‐19 pandemic: a systematic review. Telemed J E Health. 2024;30(5):1205‐1220. 38227387 10.1089/tmj.2023.0588 PMC12868971

[52] Vakkalanka JP , Santos Leon E , Davis J , Williams C , Casteel C . Examining contextual differences in suicide by rural‐urban designation and military status, 2009‐2019: a cross‐sectional analysis of the National Violent Death Reporting System. Inj Prev. 2025;31(2):121‐127. 40011043 10.1136/ip-2024-045430 PMC13075480

