# Supplementary material for: County‐level vulnerability is associated with mental health and substance use treatment among rural suicide decedents: A national multi‐year cross‐sectional study
Source: J Rural Health. 2025 Nov 6;41(4):e70094. doi: 10.1111/jrh.70094 (PMC12592758; doi:10.1111/jrh.70094)

**Supplemental Content – Table of Contents**

Appendix A. States contributing to the NVDRS by year, 2013-2022.

Appendix B. County-level Characteristics by rurality of residence among US suicide decedents.

Appendix C. Flow Chart of Analytical Sample, NVDRS 2013-2022.

| **Appendix A. States contributing to the NVDRS by year, 2013-2022.** | | | | | | | | | | |
| --- | --- | --- | --- | --- | --- | --- | --- | --- | --- | --- |
| **State** | **2013** | **2014** | **2015** | **2016** | **2017** | **2018** | **2019** | **2020** | **2021** | **2022** |
| Alabama |  |  |  |  | ^c^ | X | X | X | X | X |
| Alaska | X | X | X | X | X | X | X | X | X | X |
| Arizona |  | ^c^ | X | X | X | X | X | X | X | X |
| Arkansas |  |  |  |  |  | ^c^ | X | X | X | X |
| California |  |  |  | ^c^ | X^a^ | X^a^ | X^a^ | X | X | X^a^ |
| Colorado | X | X | X | X | X | X | X | X | X | X |
| Connecticut |  | ^c^ | X | X | X | X | X | X | X | X |
| Delaware |  |  |  | ^c^ | X | X | X | X | X | X |
| Washington DC |  |  |  | ^c^ | X | X | X | X | X | X |
| Florida |  |  |  |  |  |  | ^c^ | X | X | X^a^ |
| Georgia | X | X | X | X | X | X | X | X | X | X |
| Hawaii |  | ^c^ | X | X | ^b^ | ^b^ | X | ^b^ | ^b^ | X |
| Idaho |  |  |  |  |  |  | ^c^ | X | X | X |
| Illinois |  |  | ^c^ | X^a^ | X^a^ | X^a^ | X^a^ | X | X | X |
| Indiana |  |  | ^c^ | X | X | X | X | X | X | X |
| Iowa |  |  | ^c^ | X | X | X | X | X | X | X |
| Kansas |  | ^c^ | X | X | X | X | X | X | X | X |
| Kentucky | X | X | X | X | X | X | X | X | X | X |
| Louisiana |  |  |  |  | ^c^ | X | X | X | X | X |
| Maine |  | ^c^ | X | X | X | X | X | X | X | X |
| Maryland | X | X | X | X | X | X | X | X | X | X |
| Massachusetts | X | X | X | X | X | X | X | X | X | X |
| Michigan |  | X | X | X | X | X | X | X | X | X |
| Minnesota |  | ^c^ | X | X | X | X | X | X | X | X |
| Mississippi |  |  |  |  |  |  | ^c^ | X | X | X |
| Missouri |  |  |  |  | ^c^ | X | X | X | X | X |
| Montana |  |  |  |  |  | ^c^ | X | X | X | X |
| Nebraska |  |  |  |  | ^c^ | X | X | X | X | X |
| Nevada |  |  |  | ^c^ | X | X | X | X | X | X |
| New Hampshire |  | ^c^ | X | X | X | X | X | X | X | X |
| New Jersey | X | X | X | X | X | X | X | X | X | X |
| New Mexico | X | X | X | X | X | X | X | X | X | X |
| New York |  | ^c^ | X | X | X | ^b^ | X | X | X | X |
| North Carolina | X | X | X | X | X | X | X | X | X | X |
| North Dakota |  |  |  |  |  | ^c^ | X | X | X | X |
| Ohio | X | X | X | X | X | X | X | X | X | X |
| Oklahoma | X | X | X | X | X | X | X | X | X | X |
| Oregon | X | X | X | X | X | X | X | X | X | X |
| Pennsylvania |  |  | ^c^ | X^a^ | X^a^ | X^a^ | X^a^ | X^a^ | X^a^ | X |
| Rhode Island |  |  |  | ^c^ | X | X | X | X | X | X |
| South Carolina | X | X | X | X | X | X | X | X | X | X |
| South Dakota |  |  |  |  |  |  | ^c^ | X | X | X |
| Tennessee |  |  |  |  |  |  | ^c^ | X | X | X |
| Texas |  |  |  |  |  | ^c^ | ^b^ | X | X | X^a^ |
| Utah | X | X | X | X | X | X | X | X | X | X |
| Vermont |  | ^c^ | X | X | X | X | X | X | X | X |
| Virginia | X | X | X | X | X | X | X | X | X | X |
| Washington |  |  | ^c^ | X^a^ | X^a^ | X | X | X | X | X |
| West Virginia |  |  |  | ^c^ | X | X | X | X | X | X |
| Wisconsin | X | X | X | X | X | X | X | X | X | X |
| Wyoming |  |  |  |  |  | ^c^ | X | X | X | X |

X = Reported data for violent deaths from the entire state.

X^a^ = Reported data for violent deaths that occurred among selected counties within the state but did not include violent deaths for all counties within the state

^b^ = Excluded by Centers for Disease Control and Prevention from the NVDRS due to incomplete data reporting

^c^ = Year the state enrolled in the NVDRS

| **Appendix B. County-level Characteristics by rurality of residence among US suicide decedents.** | | |
| --- | --- | --- |
| County-level Characteristics | Rural US Decedents (n=42,021) | Urban US Decedents (n=184,188) |
|  | Median (IQR) | Median (IQR) |
| Average Number of Mentally Unhealthy Days | 4.87 (4.42-5.22) | 4.36 (4.03-4.78) |
| Percentage of Uninsured Adults | 10.03 (7.57-13.81) | 8.76 (6.48-11.84) |
| Rate of Primary Care Physicians | 50.74 (37.46-70.45) | 78.00 (57.60-100.74) |
| Rate of Population to Mental Health Providers | 163.66 (90.15-293.75) | 279.91 (171.90-362.62) |
| Percentage of Unemployed Adults | 3.99 (3.28-4.90) | 3.51 (2.93-4.09) |
| Income Inequality Ratio^1^ | 4.39 (4.06-4.79) | 4.48 (4.08-4.89) |
| Rate of Social Associations | 11.81 (9.28-14.35) | 9.02 (7.18-10.95) |
| Percentage of Adults Driving Alone during a Long Commute | 29.00 (20.80-35.40) | 35.90 (26.00-44.50) |

*^1^Income Inequality ratio was calculated as the 80^th^ income percentile / the 20^th^ income percentile.*

**Appendix C. Flow Chart of Analytical Sample, NVDRS 2013-2022.**


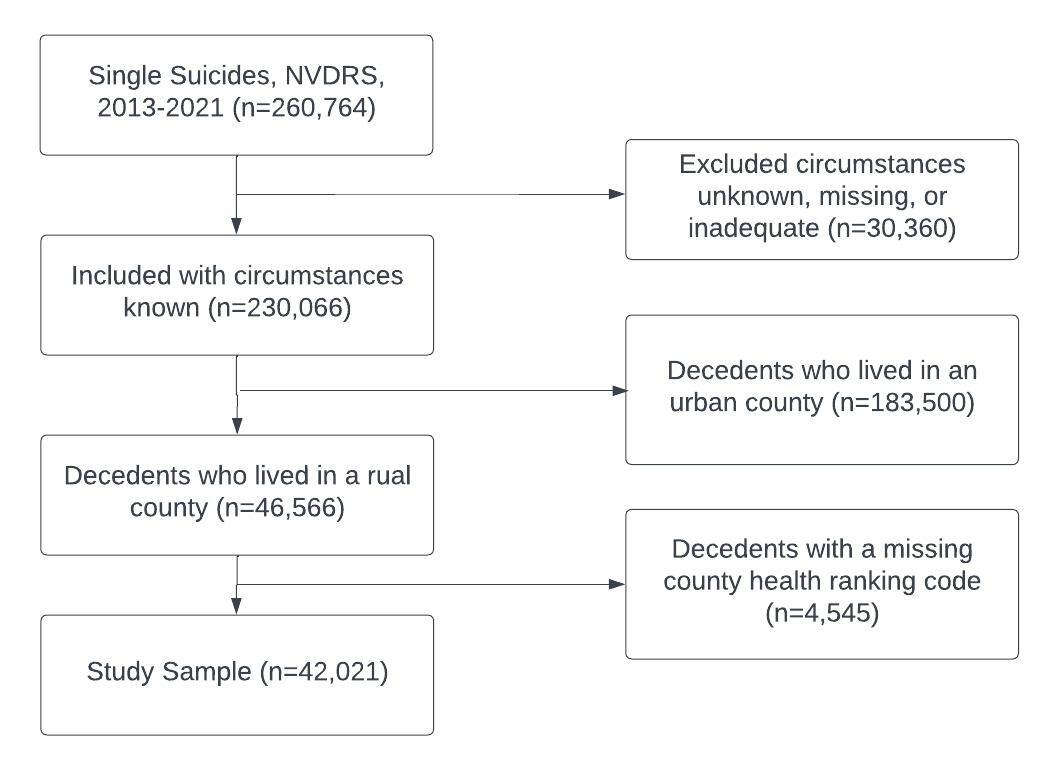

Supplement: Supplementary file 1 — Appendix A. States contributing to the NVDRS by year, 2013–2022 [file JRH-41-0-s001.docx]
